# A Cross-Sectional Investigation of the Importance of Park Features for Promoting Regular Physical Activity in Parks

**DOI:** 10.3390/ijerph14111335

**Published:** 2017-11-02

**Authors:** Sarah A. Costigan, Jenny Veitch, David Crawford, Alison Carver, Anna Timperio

**Affiliations:** 1 School of Exercise and Nutrition Sciences, Deakin University, Geelong 3220, Australia; alison.carver@acu.edu.au; 2 Institute for Physical Activity and Nutrition (IPAN), Deakin University, Geelong 3220, Australia; jenny.veitch@deakin.edu.au (J.V.); david.crawford@deakin.edu.au (D.C.); anna.timperio@deakin.edu.au (A.T.); 3 Mary MacKillop Institute for Health Research, Australian Catholic University, Melbourne 3000, Australia

**Keywords:** physical activity, park features, park use, adults

## Abstract

Introduction: Parks in the US and Australia are generally underutilised, and park visitors typically engage in low levels of physical activity (PA). Better understanding park features that may encourage visitors to be active is important. This study examined the perceived importance of park features for encouraging park-based PA and examined differences by sex, age, parental-status and participation in PA. Methods: Cross-sectional surveys were completed by local residents (*n* = 2775) living near two parks (2013/2015). Demographic variables, park visitation and leisure-time PA were self-reported, respondents rated the importance of 20 park features for encouraging park-based PA in the next fortnight. Chi-square tests of independence examined differences in importance of park features for PA among sub-groups of local residents (sex, age, parental-status, PA). Results: Park features ranked most important for park-based PA were: well maintained (96.2%), feel safe (95.4%), relaxing atmosphere (91.2%), easy to get to (91.7%), and shady trees (90.3%). All subgroups ranked ‘well maintained’ as most important. Conclusions: Natural and built environment features of parks are important for promoting adults’ park-based PA, and should be considered in park (re)design.

## 1. Introduction

Increasing opportunities to be physically active is an important priority for public health [1]. Public parks represent a key setting in the community for the promotion of physical activity across the lifespan, by providing a convenient and low-cost setting to facilitate engagement in a range of activities [2,3]. Parks can provide opportunities for physical activity, as a destination that can be reached by walking or cycling, as well as a venue for engaging in physical activity; therefore, the provision of suitable facilities and amenities within parks has the potential to support and encourage physical activity [4].

Park availability is frequently reported to be associated with increased physical activity [5,6]. For example, in a cross-sectional study examining self-reported park visitation and physical activity, each additional park visit was associated with a 26% increased likelihood of adults engaging in higher amounts of walking [7]; and, in a study of 248 adults examining parks in the United States, objectively measured park use was associated with increased moderate and moderate to vigorous physical activity (383 activity counts/min) [8]. However, despite the potential of parks as a setting for physical activity, parks are generally underutilised [8]; a recent review of international studies (predominately in the US and Australia) investigating park-based physical activity demonstrated that park visitors are not necessarily active in parks, with most park visitors engaging in only low levels of physical activity [9]. Similarly, a recent study of approximately 5000 park visitors in Melbourne, Australia found that around 62% of park visitors were sedentary (lying or sitting) or standing, with only 29% engaging in moderate-intensity physical activity and around 9% engaging in vigorous-intensity physical activity [10].

Improving environments to encourage physical activity is a widely supported method to facilitate healthier communities [11], yet a limited number of studies have reported certain park features to be associated with park-based physical activity among adults. For example, the following park features were shown to be positively associated with self-reported park-based physical activity among a sample of 1305 Danish adults: a walking/cycling route; a wooded area; a water feature; lights along trails; a pleasant view to the outside of the urban green space; a bike rack; and car parking. In addition, Kaczynski and colleagues reported the presence of a playground, sporting grounds (e.g., basketball court, tennis court, skate park) and fitness stations to be positively associated with park-based physical activity among a sample of 893 adults in the US [12]. A qualitative review of 21 studies examining park features associated with park use and physical activity highlighted attributes such as safety, aesthetics, amenities, maintenance, and proximity as being important [13]. The number of features present in a park has also been found to be positively associated with park-based physical activity [14]. However, the importance of park features which support physical activity is likely to differ among population sub-groups [6,12,13], for example, one study found considerable differences in associations between park features and physical activity according to gender, race, age and income [12].

To our knowledge, however, no studies have examined perceptions of which park features are important for park users and non-users to engage in physical activity or examined potential differences between particular sub groups, such as those with or without children or according to levels of physical activity. Identifying park features that would facilitate park visitors across a range of demographic groups to engage in physical activity will help maximise opportunities for park-based physical activity [2]. The aim of this study is therefore to examine the importance of park features for encouraging regular physical activity in parks and to investigate if differences are observed according to sex, age, parental status and levels of physical activity.

## 2. Methods

This study was nested within the Recording and EValuating Activity in a Modified Park (REVAMP) study. The methods have been described in detail previously [15]. Briefly, the REVAMP study was designed to evaluate the impact of the park modification by using multiple measures to comprehensively assess park visitation and park-based physical activity in two metropolitan parks in Melbourne, Australia: an intervention park and a control park. The intervention park (329 hectares) was located 28 km north-west of Melbourne’s central business district (CBD) in a low socio-economic status (SES) area. The control park (120 hectares) was located 22 km east of Melbourne’s CBD in a high SES area.

This study utilised cross-sectional data from self-reported resident surveys completed by adults living near the two parks in April–May 2013 (T1) and April–May 2015 (T3). Surveys were distributed via: (1) families with children attending pre-schools, primary and secondary schools located within 3 km of each park; and (2) a mail-out to households located within 5 km of each park. Overall, 1487 surveys were returned completed at T1 (15.5% response rate) and 1451 were returned completed at T3 (15.3% response rate). Due to the repeat cross-sectional design, respondents who were identified as responding at both time points (*n* = 163) were removed from the T3 sample, leaving a total sample of 2775 unique participants (T1 *n* = 1487; T3 *n* = 1288).

Ethics approval was obtained from the Deakin University Human Ethics Advisory Group (HEAG-H 46_2012), the Department of Education and Early Childhood Development (2012-001790) and the Catholic Education Office Melbourne (GE11/0009).

### 2.1. Survey Items

Demographic variables (Table 1) included age, sex, country of birth (Australia, China, Greece, India, Italy, Malta, UK or Ireland, Vietnam, other), marital status (married/de facto, separate/widowed/divorced, never married), employment status (working full-time, working part-time, unemployed, household duties and/or raising children full-time, studying, retired), highest level of education (never attended school, primary school, some high school, completed high school, technical or trade school certificate/apprenticeship, university or tertiary qualification), child aged <2 years in household (yes/no), child aged 2–15 years living in household (yes/no), child’s age (derived from parent-reported date of birth), dog ownership (yes/no), number of years lived in the neighbourhood and motor vehicle access (yes/no).

Park visitation was assessed using two items: “Have you visited a park in the past 7 days?” (response options: yes/no) and “In the past 3 months, on average, how often have you visited a park?” (response options: (1) daily; (2) 2–3 times per week; (3) once per week; (4) 2–3 times per month; (5) once per month; (6) <once per month; and (7) first time to this park). Responses were dichotomised as ‘at least once per week’ versus ‘less than once per week’.

Leisure-time physical activity (LTPA) was assessed using self-reported LTPA in the last seven days using the long form of the International Physical Activity Questionnaire (IPAQ-L). The IPAQ-L has shown excellent test-retest reliability and acceptable validity in adults [16]. Participants’ responses were dichotomized as ‘<150 min/week’ vs. ‘≥150 min/week’ to reflect the Australian Physical Activity Guidelines [17].

### 2.2. Importance of Park Features for Encouraging Park-Based Physical Activity

The importance of 20 park features for encouraging park-based physical activity was assessed by asking respondents: ‘If you were going to do regular physical activity at a park in the next two weeks, how important would each of the following features be?’ A list of features encompassing distance, amenities, aesthetics, maintenance and safety (full list of features are listed in Table 2), was developed based on our previous research and items from existing surveys [18,19,20]. Responses were based on a 5-point scale—1 = not at all important to 5 = very important—and were dichotomized as ‘not important’ (not at all important/not very important/neither) versus ‘important’ (quite important/very important). Respondents were also given the opportunity to list any other features they considered important.

### 2.3. Reliability of Survey Items

To examine test-retest reliability of the survey items, 200 reliability surveys were mailed to residents living near the control park who had already returned a completed survey; 126 surveys were returned (63% response rate). Reliability of the item examining the importance of park features for encouraging park-based physical activity was examined using one-way single measure intra-class correlation coefficient for continuous variables (ICC). An ICC of 0.75 was considered excellent and 0.4–0.74 was considered good [21]. All items but one (‘easy to get to’ ICC of 0.36) demonstrated good test-retest reliability (ICC ≥ 0.40) ranging from 0.41 to 0.72 which indicate they were acceptable for use.

### 2.4. Analyses

Descriptive characteristics were examined using SPSS 23.0 (SPSS Inc., Chicago, IL, USA). The percentage of respondents who rated each feature as important was calculated for the whole sample and for the following sub-groups: sex (males vs. females), age (≤60 years vs. >60 years), parental status (children <15 years vs. no children <15 years) and physical activity levels (<150 min LTPA vs. ≥150 min LTPA). Each park feature was then assigned a ranking from 1 (the feature with highest percentage of respondents reporting it was important) to 20 (feature with lowest percentage of respondents reporting it was important). Chi-square tests of independence examined differences in importance of park features for park-based physical activity according to the sub-groups. The level of statistical significance was set at *p* < 0.05.

## 3. Results

A profile of the survey participants is presented in Table 1. More than half (53.7%) of respondents reported usually visiting a park at least once per week in the last three months, and 58.5% reported visiting a park in the last seven days. Respondents reported participating in a mean of 212.4 (305.6) minutes of LTPA and 59.3% reported participating in at least 150 min of weekly LTPA in the last seven days.

### 3.1. Importance of Park Features for Encouraging Park-Based Physical Activity

Overall, the five features most commonly considered to be important for encouraging park-based physical activity were: it is well maintained (96.2%), you personally feel safe going there whenever you want to (95.4%), it has a relaxing atmosphere (91.2%), it is easy to get to (91.7%), and there are shade trees (90.3%). Whereas, the park features least commonly reported as important for encouraging park-based physical activity included presence of bike racks (49.9%), there is a dog off-leash area (42.5%) and it is close to public transport (28.3%). Consistent with the overall sample, being ‘well maintained’ was the park feature most commonly reported as important for encouraging physical activity, in all sub-groups, followed by ‘you personally feel safe going there’ for six of eight sub-groups.

Although similarities in ranked order were identified for the importance of features (from most to least common), numerous differences by sub-group in the proportion of respondents who perceived specific park features as important for encouraging park-based physical activity in the next fortnight were identified (see Table 2). In comparison to men, a greater percentage of women considered 14 of the 20 features to be more important, while a higher percentage of men considered closeness to public transport to be important. Compared to those aged <60 years, a higher percentage of older adults considered trees and birdlife, benches, attractive features and closeness to public transport to be important. A higher percentage of parents of children aged <15 years considered maintenance, feeling safe, ease of access/close to home, play equipment, bike racks, toilets and interesting walks to be important, compared to respondents without children <15 years. Finally, a higher percentage of those who met the physical activity guidelines considered interesting walks/cycles/jogs, a variety of paths, drinking fountains and attractive features as important compared to those not meeting the guidelines.

### 3.2. Additional Features

In addition to the 20 park features listed, a small number of respondents (*n* = 409; 14.7%) reported ‘other park features’ would be important for engaging in physical activity in the next two weeks. These included: rubbish bins (10.3%), BBQ’s/picnic areas (10.3%), well-maintained paths/tracks (7.6%), well-maintained play equipment (7.1%), availability of fitness stations (5.9%), dog-free areas (4.9%), specific facilities for dogs (e.g., water, fenced areas, waste disposal bins) (4.9%), lighting (4.4%), and well-maintained toilets (3.2%).

## 4. Discussion

To encourage park-based physical activity, it is important to identify specific park features which may encourage park visitors to engage in physical activity during their park visit. The current study examined the importance of various natural and built park features for encouraging regular park-based physical activity among adults and differences in the importance of these features according to sex, age, parental status and activity level. Overall, park maintenance, feeling safe at the park, a relaxing atmosphere, ease to get to and shade were the features most commonly perceived as important for encouraging park-based physical activity in the next two weeks. These five park features ranked similarly (according to prevalence) within each sub-group with the exception of respondents aged ≥60 years who ranked ‘trees and birdlife’ higher than it being ‘easy to get to’. Our findings are consistent with a qualitative review of 21 studies examining park features associated with park use and physical activity, which found park features including safety, aesthetics, amenities, and maintenance were important [13].

Park features that were less commonly reported as important for encouraging park-based physical activity in the next two weeks included car parking, attractive features, bike racks, an off-leash dog area and closeness to public transport. These five park features were ranked among the least common by all sub-groups, with the exception of respondents aged ≥60 years and respondents without children. It is important to consider, however, that the feature least commonly reported as important (close to public transport) was still considered important by 28% of the sample. Our findings regarding bike racks and public transport may be reflective of public transport and bikes not being common transport modes among this sample. For instance, in the current study, 95.3% reported owning a car. This finding is consistent with data from park intercept interviews collected with park visitors as part of the REVAMP study, where 90% of respondents reported using inactive modes of transport to travel to the parks [22].

Furthermore, the presence of off-leash areas for dogs was reported as important for encouraging park-based physical activity by a lower percentage (42.5%, ranked 19) of respondents, which may suggest that, while some adults perceived this feature as important for being active, other park features such as access to interesting walks and jogs (overall ranked 7th) and a variety of paths (overall ranked 12th) could also provide opportunities to be active whilst simultaneously exercising a dog, compared to an off-leash area. As reported in an observational study of dog walking and physical activity [23], a higher frequency and duration of walking in parks was self-reported by dog owners in comparison to park attendees who visited without a dog.

While the ranked importance of the 20 built and natural park features examined did not differ greatly between sub-groups, there were some significant differences between sub-group categories in the importance of park features for engaging in physical activity. In comparison to men, a higher percentage of women reported 14 of 20 park features to be important for park-based physical activity. A study conducted by Cohen et al. [6], which included systematic observations at parks and resident/park user interviews (*n* = 1318) found that when women visited parks, they were more likely to spend time in areas such as playgrounds to supervise children. Women’s role in supervising children at parks may help explain why features such as safety, ease of access, friendly people, drinking fountains and toilets were identified as important for engaging in park-based physical activity, as opportunities for women to be active may be reliant on being able to simultaneously supervise and cater for their child’s needs. Therefore, providing features that increase opportunities for adults, particularly women, to be active near park facilities that attract children (such as playgrounds) may be important (e.g., fitness stations near playground).

In comparison to adults aged <60 years, a higher proportion of respondents aged ≥60 years reported trees and bird life, benches, and attractive features as important for encouraging park-based physical activity. It may be that environmental features encourage older adults to visit a park and then whilst at the park they may be more likely to engage in physical activity (e.g., walking); whereas benches may be necessary for rest during walks or other activities. For older adults, parks are key settings for relaxation and mindfulness activities [24] and may play a role in slowing age-related declines in physical activity levels [25]. Our findings are consistent with a number of studies examining environmental influences on walking, which have reported significant positive associations between aesthetically pleasant environments and walking for exercise/recreation [26,27,28]. However, these studies did not specifically examine older adults, and only included walking behaviours, and not overall physical activity in parks. Future investigation should examine the relationship between park features and physical activity for specific age groups.

A higher percentage of respondents participating in >150 min/week LTPA (compared to lower weekly LTPA) reported interesting walks, cycles or jogs to do, a variety of paths and drinking fountains as important for encouraging park-based physical activity, which may reflect that active respondents are already using these features for physical activity and recognize they are important. Interestingly, a study of adults 45–65 years (*n* = 279) examining how natural environments were related to physical activity, found that larger-sized (compared to smaller-sized) natural environments (e.g., parks) were associated with increased MVPA, walking, jogging and cycling [29], which may be explained by larger spaces providing more facilities to encourage physical activity (e.g., walking trails, paths, sports) [27]. Of the respondents completing the questionnaire, approximately 14% suggested ‘other park features’ that they considered would be important for encouraging park-based activity. Suggestions mostly related to the provision of “well-maintained” park features such as toilets, play equipment, and paths/tracks, as well as lighting and rubbish bins. Contrary to expectations, barbeque and picnic areas were reported to be relatively important for encouraging park-based physical activity (by 10.4% of those reporting ‘other park features’); however, Jansen et al. [29] reported recreational spaces such as picnic areas mostly facilitate light physical activity (e.g., small games) in Dutch adults aged 45–65. The inclusion of fitness stations was suggested by 5.9% as being important for encouraging park-based physical activity. The presence of fitness stations has been previously reported to be associated with park-based physical activity among female adults aged 18–39 years [12] and for seniors; however, training programs may be required to instruct new users and encourage their use of such equipment [30].

Understanding the importance of park features for physical activity is necessary to ensure parks are designed to maximise opportunities for people across a range of demographic groups to be active when visiting a park, and is potentially a long-term and sustainable way to increase physical activity for the general population. Strengths of this study include the large sample size and diverse mix of demographic characteristics. Respondents were recruited from both high and low socio-economic areas, and included regular and irregular park visitors. However, it is important to acknowledge that these study findings are reflective of participants living nearby two large parks located in Melbourne, Australia, which may limit the ability to generalise results to people living in other areas of Australia or internationally. Other limitations include the use of self-report measures, which may be subject to recall error—for example, over/under-reporting LTPA—or misinterpretation of questions. The categories of ‘quite important’ and ‘very important’ were collapsed in this study, which prevented the ability to examine the degree of importance. In addition, participants were not asked to ‘rank’ each feature based on importance, ranking of features was completed post-hoc based on prevalence (%). Overall survey response rates were also relatively low, which may limit the generalisability of study findings. Finally, participants were only asked to report on 20 specific park features, and other park features not listed may also be important for encouraging park-based physical activity (for example 14.7% of respondents provided a response to ‘other park features’).

These findings are important, as they could inform local councils and park planners when designing new parks or redeveloping existing parks. While our study considered the importance of features for a range of population sub-groups, future studies are encouraged to examine the importance of park features for population groups specifically at risk of low levels of physical activity, for example, those with disabilities, culturally and linguistically diverse groups, rural populations and minority groups [31]. It may also be useful to conduct more in-depth interviews with those who are inactive in parks to investigate what would help them to be active, and to examine if other factors apart from physical features are important for physical activity (for example social factors, having others to be active with, feeling safe).

## 5. Conclusions

This study contributes to the evidence base exploring the importance of park features for promoting and encouraging park-based physical activity among adults. The findings indicate that both natural and built park features are important for encouraging and promoting physical activity. Whilst we examined the importance of park features according to sex, age, parental status, and physical activity, the ranked importance of park features remained relatively consistent across groups.

The findings of this study highlight park features that would facilitate adult park visitors’ (across a range of demographic groups) engagement in park-based physical activity. Our findings suggest parks that are well maintained, safe, relaxing, easy to get to, and have shady trees may be particularly important for encouraging park-based physical activity among adults and should be considered in park (re)design guidelines. To promote park-based physical activity across the lifespan and for different user groups [27], provision and maintenance of park features that are considered important by a variety of park-users is necessary.

## Figures and Tables

**Table 1:** Demographics of questionnaire respondents (*n* = 2775).

Demographic Characteristic	Mean (SD) or %
Age in Years (Mean (sd))	51.13 (14.68)
Sex (%)	
Male	32.6%
Female	67.4%
Country of birth (%)	
Born in Australia	59.0%
Born elsewhere	41.0%
Marital status (%)	
Married/defacto	79.5%
Separated/widowed/divorced	14.0%
Never married	6.4%
Employment status (%)	
Working full-time	33.2%
Working part-time	23.9%
Unemployed/keeping house/raising children/studying	22.1%
Retired	20.9%
Education level (%)	
No formal qualifications	13.0%
Year 12/apprentice/diploma or certificate	30.3%
University degree or higher degree	56.7%
Children ≤ 15 years (%)	60.9%
Child age (mean (sd))	8.37 (3.6)
Dog ownership (%)	32.9%
Years lived in neighbourhood (mean (sd))	18.0 (15.022)
Do you have access to a motor vehicle for private use? (%)	
Yes	95.3%
No	4.7%
Park Location (%)	
Intervention	46.2%
Control	53.8%
Park Visitation (%)	
Visited a park in the past 7 days	
Yes	58.5%
No	41.5%
Frequency of visitation in the past 3 months (%)	
Less than once per week	46.3%
At least once per week	53.7%
Leisure time physical activity	
Participate in more than 150 min/week	59.3%
Minutes/week of leisure time physical activity; mean (SD)	212.42 (305.59)

**Table 2:** The importance (%) and ranking of park features for engaging in physical activity in the next two weeks.

Park Feature	Total	Sex		Age		Parental Status		Physical Activity Levels	
	*n* = 2775 % (Ranking)	Male *n* = 897 % (Ranking)	Female *n* = 1856 % (Ranking)	<60 Years *n* = 1964 % (Ranking)	≥60 Years *n* = 745 % (Ranking)	No Children < 15 Years *n* = 1392 % (Ranking)	Child(ren) <15 Years *n* = 1397 % (Ranking)	<150 min/week *n* = 744 % (Ranking)	≥150 min/week *n* = 550 % (Ranking)
Well maintained	96.2 (1)	94.9 (1) *	96.9 (1)	97.3 (1) **	93.7 (1)	95.1 (1) *	97.3 (1)	96.1 (1)	97.2 (1)
Feel safe going there	95.4 (2)	92.5 (2) **	96.8 (2)	97.0 (2) **	91.1 (3)	93.4 (2) **	97.3 (1)	95.1 (2)	96.1 (2)
Relaxing	91.2 (3)	90.3 (3)	91.5 (4)	91.3 (4)	91.1 (4)	91.8 (3)	90.6 (5)	90.5 (3)	91.2 (5)
Easy to get to	90.7 (4)	85.8 (5) **	93.1 (3)	94.0 (3) **	82.0 (10)	87.0 (5) **	94.3 (2)	91.8 (4)	93.5 (3)
Shade trees	90.3 (5)	88.9 (4)	91.0 (5)	89.9 (5)	91.2 (2)	89.1 (4)	91.4 (4)	90.1 (5)	91.8 (4)
Friendly people	86.6 (6)	83.0 (7) **	88.4 (6)	88.0 (6) **	82.6 (8)	85.4 (6)	87.8 (6)	85.8 (6)	87.9 (7)
Interesting walks/cycles/jogs	86.0 (7)	83.6 (6) *	87.3 (7)	87.2 (7) **	82.9 (7)	84.7 (8) *	87.3 (7)	82.6 (7) **	91.2 (6)
Toilets	83.3 (8)	80.4 (9) **	84.8 (8)	83.9 (8)	81.8 (11)	81.8 (11) *	84.8 (8)	81.4 (8)	84.3 (10)
Trees and birdlife	83.2 (9)	82.5 (8)	83.5 (10)	82.2 (10) *	86.2 (5)	85.1 (7) **	81.3 (9)	82.7 (9)	84.9 (9)
Play equipment	81.5 (10)	77.0 (12) **	83.7 (9)	83.8 (9) **	75.8 (13)	71.2 (16) **	91.5 (3)	83.5 (10) *	78.5 (14)
Benches	81.0 (11)	78.1 (11) *	82.4 (11)	79.4 (13) **	84.6 (6)	83.8 (9) **	78.4 (11)	82.0 (11)	80.6 (12)
Variety of paths	80.4 (12)	79.1 (10)	81.1 (14)	79.9 (12)	82.2 (9)	83.1 (10) **	77.8 (13)	77.1 (12) **	87.2 (8)
Walking distance from home or work	79.2 (13)	74.2 (13) **	81.8 (12)	82.0 (11) **	71.8 (14)	77.6 (13) *	80.8 (10)	81.0 (13)	82.0 (11)
Other people using it	78.3 (14)	71.8 (14) **	81.4 (13)	78.2 (14)	78.3 (12)	78.6 (12)	78.1 (12)	77.3 (14)	80.0 (13)
Drinking fountains	73.1 (15)	69.0 (15) **	75.1 (15)	73.7 (15)	70.8 (16)	72.2 (14)	74.0 (14)	70.8 (15) *	76.9 (15)
Car parking	72.6 (16)	67.4 (16) **	75.0 (16)	73.4 (16)	71.5 (15)	71.5 (15)	73.6 (15)	74.9 (16)	71.8 (16)
Attractive features	60.6 (17)	62.8 (17)	59.6 (17)	59.2 (17) *	64.6 (17)	66.6 (17) **	54.8 (16)	56.8 (17) *	63.9 (17)
Bike racks	49.9 (18)	42.1 (18) **	53.7 (18)	52.4 (18) **	44.2 (19)	46.0 (19) **	53.7 (17)	50.3 (18)	52.4 (18)
Off-leash area	42.5 (19)	37.8 (19) **	44.7 (19)	41.7 (19)	44.3 (18)	46.7 (18) **	38.5 (18)	40.6 (19)	43.9 (19)
Close to public transport	28.3 (20)	31.4 (20) *	26.7 (20)	24.5 (20) **	38.0 (20)	36.4 (20) **	20.6 (19)	28.2 (20) *	22.8 (20)

* *p* < 0.05 ** *p* < 0.01; % indicating feature was important; ranking from highest (1) to lowest (20).
